# The Potential of Hormonal Therapies for Treatment of Triple-Negative Breast Cancer

**DOI:** 10.3390/cancers15194702

**Published:** 2023-09-24

**Authors:** Melanie Kirkby, Alyanna M. Popatia, Jessie R. Lavoie, Lisheng Wang

**Affiliations:** 1 Department of Biochemistry, Microbiology and Immunology, Faculty of Medicine, University of Ottawa, 451 Smyth Road, Ottawa, ON K1H 8M5, Canada; mkirk071@uottawa.ca (M.K.); apopa084@uottawa.ca (A.M.P.); 2 Ottawa Institute of Systems Biology, University of Ottawa, 451 Smyth Road, Ottawa, ON K1H 8M5, Canada; 3 The Centre for Infection, Immunity, and Inflammation (CI3), University of Ottawa, 451 Smyth Road, Ottawa, ON K1H 8M5, Canada; 4 Centre for Oncology, Radiopharmaceuticals and Research, Biologic and Radiopharmaceutical Drugs Directorate, Health Products and Food Branch, Health Canada, Ottawa, ON K1A 0K9, Canada; 5 Regenerative Medicine Program, Ottawa Hospital Research Institute, Ottawa, ON K1H 8L6, Canada

**Keywords:** triple-negative breast cancer, hormone therapy, estrogen receptor beta, androgen receptor, glucocorticoid receptor, tamoxifen, enzalutamide, mifepristone

## Abstract

**Simple Summary:**

The presence of estrogen receptor beta, the androgen receptor, and the glucocorticoid receptor on triple-negative breast cancer cells has opened the possibility for the development of new treatment approaches against this disease. This review summarizes the current knowledge and development regarding the presence and function of these receptors in triple-negative breast cancer. Key data from current and previous clinical trials targeting these receptors are also described in detail.

**Abstract:**

Triple-negative breast cancer (TNBC) is considered one of the most aggressive forms of breast cancer with poor survival rates compared to other breast cancer subtypes. TNBC is characterized by the absence of the estrogen receptor alpha, progesterone receptor, and the human epidermal growth factor receptor 2, limiting those viable treatment options available to patients with other breast cancer subtypes. Furthermore, due to the particularly high heterogeneity of TNBC, conventional treatments such as chemotherapy are not universally effective, leading to drug resistance and intolerable side effects. Thus, there is a pressing need to discover new therapies beneficial to TNBC patients. This review highlights current findings regarding the roles of three steroid hormone receptors, estrogen receptor beta, the androgen receptor, and the glucocorticoid receptor, in the progression of TNBC. In addition, we discussed several ongoing and completed clinical trials targeting these hormone receptors in TNBC patients.

## 1. Introduction

**Figure 1:**
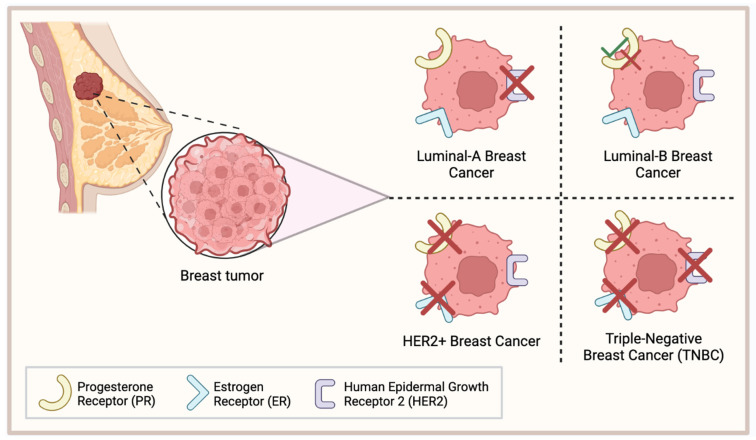
Classification of common breast cancers. Luminal-A breast cancer lacks expression of HER2; Luminal-B breast cancer is either PR+/−; HER2+ breast cancer lacks PR and ER expression; triple-negative breast cancer (TNBC) lacks expression of PR, ER, and HER2. This figure was made using Biorender.com.

Breast cancer is one of the most lethal and complex diseases that have resulted in the deaths of millions worldwide [1]. Approximately 15% of all breast cancer cases are classified as triple-negative breast cancer (TNBC) which, relative to the other subtypes, has the most aggressive phenotype, the worst overall survival (OS), and a higher occurrence of metastases at the time of diagnosis [2,3]. TNBC is classically defined by a lack of the hormone receptor estrogen receptor alpha (ERα) and the progesterone receptors (PRs) and by an absence of human epidermal growth factor receptor 2 (HER2) [4] (Figure 1). As a result, TNBC tumors are not susceptible to the targeted therapies that have been developed for other breast cancer subtypes and TNBC patients most commonly rely on chemotherapy for treatment.

TNBC is considered a fairly heterogenous disease, where four distinct TNBC subtypes have been identified based on their unique gene expression profiles: basal-like 1 (BL1), basal-like 2 (BL2), mesenchymal (M), and luminal androgen receptor (LAR) [5,6]. Each subtype is associated with a unique clinical profile and a differing response to adjuvant and neoadjuvant chemotherapy. Particularly, the BL1 classification was associated with a greater response to chemotherapy and a longer relapse-free survival period [6]. Conversely, the BL2 and LAR phenotypes were more resistant to neoadjuvant chemotherapy and only 18% and 29% of patients achieved a pathological complete response, respectively [6,7]. Thus, in part due to the heterogeneity of this disease, there is a lack of viable treatment options universally available for TNBC patients. This is reflected by TNBC’s poor prognosis, where the five-year survival rate for patients with metastatic TNBC is only around 10% [2]. Additionally, approximately 40% of stage I–III TNBC patients will experience relapse following treatment, with the greatest risk present during the first three years post-therapy [3,8]. Thus, there is a critical need to develop novel therapies against this disease.

The aim of this review is to highlight three possible hormone receptors that could be used clinically for TNBC patients. Specifically, we discuss the current research surrounding the role of estrogen receptor beta (ERβ), the androgen receptor (AR), and the glucocorticoid receptor (GR) in the progression of TNBC, as well as their implications on survival and treatment. We also highlight several clinical trials targeting these hormone receptors in TNBC patients and the major outcomes from these studies.

## 2. Estrogen Receptor Beta

**Figure 2:**
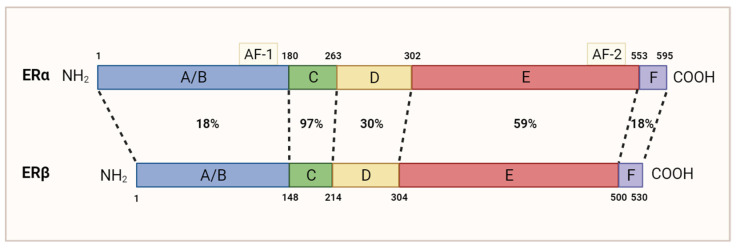
Homology between ERα and ERβ’s amino acid sequence. Dotted lines are used to compare domains with the same function. The N-terminal domain (A/B) containing AF-1 is 18% homologous. The DNA-binding domain (C) is 97% homologous. The hinge domain (D) is 30% homologous. The ligand-binding domain (E), which contains the AF-2 domain, is 59% homologous. The carboxyl-terminal domain (F) is 18% homologous. Adapted from [13]; originally published under Creative Commons Attribution 3.0 Unported (CC BY 3.0) license. Available from: 10.5772/21807. This figure was made using Biorender.com.

TNBC is commonly characterized by an absence of ERα. Despite this, approximately 5–10% of ERα-negative breast cancer tumors responded positively to treatment with anti-ERα drug tamoxifen [9,10], indicating the possibility of alternative ERα-independent signaling pathways. Growing research has associated the presence of ERβ with the outcome of various TNBC patients. Similar to ERα, ERβ is a steroid hormone receptor that binds various estrogenic compounds, including estradiol-β-17 (E2), to regulate the transcription of its downstream gene targets [11]. Independently of ERα, ERβ is encoded by the gene *ESR2* and can be spliced into five distinct isoforms, ERβ1-5; however, only the full-length variant ERβ1 is functionally capable of binding estrogenic compounds [11,12]. Although ERα and ERβ share a similar genetic identity and are composed of the same five domains, they diverge most significantly in their N-terminal region(18%), which harbor the activation function 1 (AF-1) domain, and C-terminal (18%) region (Figure 2), resulting in ER-specific gene regulation [13]. ERβ’s classical mechanism of action is functionally similar to that of ERα. In its unbound state, ERβ is bound to the chaperone protein heat shock protein 90 (HSP90) [14]. The binding of its estrogenic compound leads to dimerization of the receptor, dissociation of HSP90, and subsequent translocation to the nucleus. There, ERβ can regulate gene transcription through interactions with the estrogen response elements. ERβ is widely expressed in normal breast epithelial cells and is present in other tissue including the prostate, ovaries, and brain [15,16,17].

## 3. Estrogen Receptor Beta in the Progression of TNBC

Previously, there has been controversy regarding ERβ’s existence within diseased breast tissue. Specifically, previous reports investigating ERβ in breast cancer have used non-specific anti-ERβ antibodies, likely producing a false positive result in regard to ERβ expression [18,19,20]. Since then, several studies using validated anti-ERβ antibodies have generated different results. One study validating the use of antibody PPZ0506 for ERβ detection was unable to detect any transcriptional activity in both normal and diseased breast tissue [20]. In contrast, another study using both antibodies PPZ0506 and PPG5/10 and an optimized immunohistochemistry-based assay demonstrated that approximately 20–30% of all breast carcinomas tested positive for ERβ expression [17]. In TNBC patients specifically, the percentage of ERβ-positive tumors ranged between 25 and 83%; however, the majority of these studies used non-specific antibodies to reach these conclusions [21,22,23,24]. A recent study using the validated CWK-F12 ERβ1 antibody found that 72% of TNBC tumor samples expressed ERβ1, aligning well with studies using non-specific antibodies; however, its expression was not associated with any TNBC subtype (BL1, BL2, M, or LAR) in particular [25]. Overall, the current findings support the idea that ERβ is expressed in a significant proportion of TNBC tumors; however, further research using validated ERβ antibodies is needed.

The expression of ERβ on TNBC tumors could have several clinical implications. Interestingly, ERβ expression does not depend on the presence or absence of the classical breast cancer markers, indicating that ERβ signaling can function independently of ERα [21,26]. Although several studies have attempted to elucidate Erβ’s role in the progression of TNBC, a clear understanding has not been reached. In TNBC cell lines with inducible ERβ1 expression, cellular growth was halted through inhibition of the G1/S cell cycle transition, and this phenomenon was enhanced by the addition of E2 [27]. Furthermore, a knockdown of ERβ at the transcriptional level increased the expression of several pro-tumorigenic genes, including transforming growth factor beta (TGFβ) 1/2 [28].

In direct contrast, a growing number of studies have demonstrated that, under certain conditions, ERβ can instead promote tumor growth in TNBC. As an example, one study demonstrated that ERβ expression in ERα-negative cell lines resulted in increased insulin growth factor (IGF) 2 (IGF2) secretion, upregulation of MAPK/PI3K signaling, and was associated with a decrease in relapse-free survival [29]. The observed discrepancy could be attributed to the differential regulation imposed by ERβ isoforms beyond ERβ1. ERβ2 and ERβ5 were the predominant ERβ isoforms found in human TNBC tumors and cell lines, and an upregulation of either resulted in enhanced cell migration and invasion [30]. Conversely, overexpression of ERβ1 was associated with a suppression of tumor growth and survival. Furthermore, there is a lack of standardization in detection methods, tissue preparation, and antibody selection, as well as minimal information regarding ERβ’s role in each TNBC subtype [29]. Each of these factors could contribute to the conflicting results published so far. Thus, further clarification is needed to fully elucidate the functions of each ERβ isoform in the context of TNBC.

Several researchers have proposed that ERβ’s functioning may also depend on other signaling pathways, particularly mutations in tumor suppressor *P53*. Around 80% of all TNBC patients harbor a mutation in the *P53* gene, often resulting in a gain in oncogenic functioning [31]. Mutant p53 can form a complex with p63 and p73, inhibiting their activity and promoting cancer cell metastasis [32,33]. When ERβ is present in vitro, it can interact with mutant p53 to disrupt the complex with either p63 or p73, inhibiting tumor growth [34,35]. ERβ’s interaction results in a reconfiguration of mutant p53′s structure, returning its structure to the wildtype form and preventing its oncogenic functioning. In patients with wildtype p53, ERβ alters p53′s transcriptional regulation, resulting in a pro-proliferative phenotype [35]. Similar trends were observed in TNBC patients’ OS, where patients expressing mutant p53 and high levels of ERβ had the best outcome. Of note, the sequestration of patient phenotypes may also allow clinicians to predict the benefit of tamoxifen use in TNBC patients. Patients expressing high levels of ERβ and mutant p53 showed an increased responsiveness to tamoxifen treatment while those with wildtype p53 received little to no benefit at all. Thus, ERβ and mutant p53 could serve as useful biomarkers to predict tamoxifen’s effectiveness in TNBC patients.

## 4. Clinical Data regarding Estrogen Receptor Beta in TNBC

Because ERβ is believed to impact the progression of some TNBC tumors, growing research has looked at targeting the receptor in a clinical setting. This section describes the current clinical data available for targeting ERβ and what results have been obtained thus far. Table 1 summarizes the major findings from clinical trials targeting ERβ.

### 4.1. Selective Estrogen Receptor Modulators

Although not traditionally used for ERα-negative tumors, preclinical and clinical research has shown that ERβ can influence the effectiveness of tamoxifen in a small percentage of TNBC patients. In a 2023 case study, the use of tamoxifen in an ERβ-positive / p53-mutant TNBC patient who had experienced brain metastases was evaluated [36]. Treatment with tamoxifen led to a significant reduction in tumor volume of the brain metastases. This observation was predominantly a result of tamoxifen’s ability to increase ERβ’s interaction with mutant p53 in the cancerous cells, providing support for the clinical benefit of targeting ERβ in patients. As of today, the patient has shown no signs of disease progression. Although a larger number of patients is needed to validate this finding, this case study was the first to evaluate the status of p53 and ERβ for TNBC treatment. An ongoing phase III clinical trial (NCT02062489) is currently evaluating the effectiveness of adjuvant tamoxifen therapy in ERα/PR-negative, ERβ-positive operable breast cancer patients [37]. The primary objective of this trial is to evaluate tamoxifen’s effect on OS and disease-free survival (DFS) in tumors highly expressing ERβ and to determine if Erβ is positivity correlated with a response to estrogen therapy. Results for this study are expected in May 2026.

Toremifene is another FDA-approved nonsteroidal selective estrogen receptor modulator that has demonstrated a similar efficacy and safety profile to tamoxifen [41]. A phase IV clinical trial (NCT02089854) evaluating the use of adjuvant endocrine therapy (toremifene and anastrozole, a nonsteroidal aromatase inhibitor) in patients with operable ERβ-positive TNBC tumors is underway [38]. The effect of this endocrine therapy on DFS and OS will be evaluated to determine its effectiveness relative to the control group (i.e., no adjuvant endocrine therapy). Results for this trial have not been published yet.

### 4.2. 17β-Estradiol

E2 is the ligand for both ERα and ERβ. As previously mentioned, in TNBC cell lines with inducible expression of ERβ, treatment with E2 promoted G1 cell cycle arrest and tumor regression [27], suggesting that E2 could have clinical potential in treating TNBC. In a phase II clinical trial, the use of high-dose oral E2 was evaluated in 17 patients with metastatic TNBC, regardless of their ERβ status [39]. Among the 13 patients who expressed high levels of ERβ, only 1 patient demonstrated a partial response to E2 treatment, and treatment was shown to have little effect on OS and progression-free survival (PFS). E2 treatment was generally well tolerated by the patients. Grade 3/4 adverse events (AE) were observed in 4 of the 17 patients evaluated with two cases of grade 3 dyspnea, one case of grade 3 vomiting, and one case of grade 4 thromboembolism reported. Although the trial concluded with minimal effectiveness, the authors do not rule out the use of ERβ entirely. Improved detection of ERβ and the use of alternative ERβ agonists could result in increased clinical benefit for a subset of TNBC patients.

An ongoing phase II clinical trial by Mayo Clinic (NCT03941730) is evaluating the effectiveness of E2 in metastatic TNBC patients overexpressing ERβ [40]. No results have been posted as of this date, and completion of this study is expected in April 2024.

## 5. Androgen Receptor

**Figure 3:**
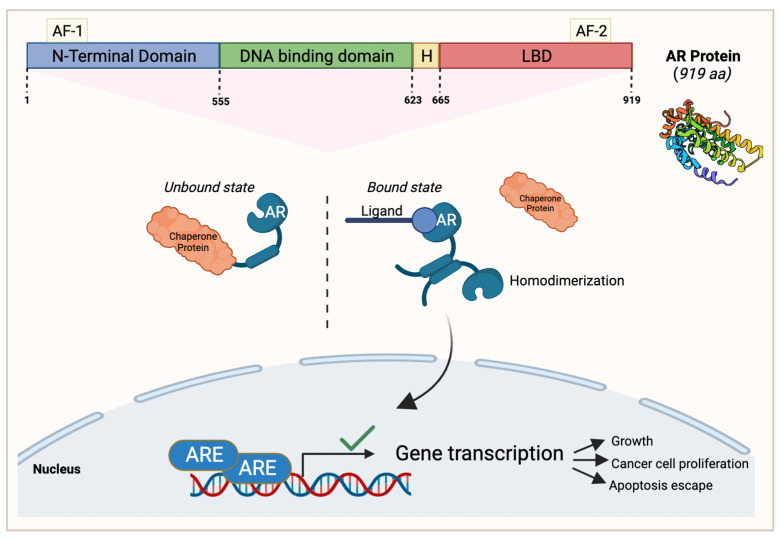
An overview of the androgen receptor (AR) protein structure and its signaling pathway in cancerous cells. AR’s N-terminal region contains androgen-independent activation function 1 (AF-1), followed by the DNA-binding domain, the hinge domain (H), and the ligand-binding domain (LBD) contained in the C-terminus with the AF-2 domain. In AR’s unbound state, it interacts with a chaperone protein. When a ligand of interest such as androgen binds to AR, it dissociates from the chaperone protein and homodimerizes. It then translocates to the nucleus to activate gene transcription that enables cell growth, cancer cell proliferation, and apoptosis escape. This figure was made using Biorender.com.

One of the most commonly overexpressed steroid nuclear receptors in breast cancer patients is the androgen receptor (AR), with a 70% occurrence leading to increased pathogenesis [42]. This receptor is a single polypeptide that is expressed in 10–43% of TNBC subtypes [43]. The AR has different domains in its structure that allow it to carry out its functions (Figure 2). The first domain is the N-terminal region which contains androgen-independent AF-1. The DNA-binding domain (DBD) interacts with incoming androgen signaling components while the hinge domain connects the DBD with the ligand-binding domain (LBD) [44]. Lastly, the C-terminal LBD binds to androgen and anti-androgen ligands contained in the C-terminus with the androgen-dependent activation function 2 domain (Figure 3).

The AR has the capacity to bind to different ligands, such as growth factors including IGF and TGFβ, or endogenous androgens [45,46]; however, when it is unbound, it will interact with chaperone proteins [42]. Once AR binds to a ligand of interest, it will dissociate from the chaperone protein and will reconfigure into a homodimer that induces target gene transcription by translocating into the nucleus and activating a series of signaling events that lead to apoptosis escape and cancer cell proliferation [42,47,48] (Figure 2). AR signaling is important for the functioning of several organs in the human body including the cardiovascular system, musculoskeletal system, prostate, and the nervous system [49].

## 6. The Role of the Androgen Receptor in TNBC

The most consistently identified subtype of TNBC that is characterized by AR mRNA and its target genes’ expression is the LAR subtype [5,43,50]. LAR TNBCs show increased resistance to both neoadjuvant and adjuvant chemotherapy and demonstrate a poor pathological complete response [7,43,51]. Although AR signaling has been implicated in AR-positive TNBC, its involvement in disease progression is not completely understood. When the LAR subtype was identified, the targeting of AR in LAR cells decreased cell proliferation [5]. TNBC cells expressing high levels of AR increased expression of genes associated with cell cycle progression when compared to AR-negative cell lines [52]. Interestingly, across all the breast cancer subtypes, AR was present on both the primary and metastatic breast carcinomas, with some metastatic tumors showing elevated AR levels [53]. In vitro analysis of TNBC cell lines demonstrated that an upregulation of AR promoted anchorage-independent survival [54], suggesting that AR expression may be essential for successful metastasis to occur. Mechanistically, the presence of androgen can trigger the formation of a complex between AR, Src, FAK, and PI3K to modulate focal adhesion and promote cellular invasion [55]. Additionally, AR was also shown to promote cancer stem cell growth, and treatment with the antiandrogen enzalutamide decreased the formation of mammospheres in vitro and reduced tumor growth in vivo [54]. Because cancer stem cells are capable of initiating tumor growth, the targeting of AR alongside chemotherapy may be a viable method for preventing recurrent disease.

Current studies investigating AR’s role as a prognostic marker in breast cancer have yielded controversial results. Several studies analyzing AR-positive and AR-negative TNBC tumors indicate that the expression of AR is associated with an increased OS and DFS [56,57,58]. However, as described above, this is in direct contrast with most experimental results obtained so far. Furthermore, a small number of studies report that AR expression is associated with an increased rate of metastasis [59,60], while others have stated that it has no effect upon OS in TNBC [61,62]. Possible explanations for this discrepancy can include several factors such as the demographic and TNBC subtypes being analyzed, as well as the effect of different AR mutations upon patient outcome [43]. A lack of standardization among the methodologies and AR cut-off percentages used could also contribute to the conflicting results [63]. Despite this, targeting AR is still a viable option as, similar to ERα-positive tumors, AR-positive tumors are dependent on AR function [54]. Therefore, therapies targeting AR are an area of great interest, particularly for the LAR TNBC subtype.

## 7. Clinical Trials Targeting the Androgen Receptor in TNBC

Because AR has been heavily implicated in the progression of AR-positive LAR and non-LAR TNBC subtypes, several researchers are now looking to target AR as a novel therapeutic avenue for TNBC patients. In this section, the results of several ongoing and recent clinical trials targeting AR are described. The results from these clinical trials are summarized in Table 2.

### 7.1. Enzalutamide

Enzalutamide is a second-generation antiandrogen that has been FDA-approved for the treatment of metastatic castration-resistant prostate cancer [71]. Enzalutamide works by binding to the ligand-binding domain of AR, inhibiting the binding of androgen ligands to its receptor. As a result, nuclear translocation and chromosomal DNA interactions are prevented, blocking the transcription of target genes and oncogenic processes. A phase II clinical trial (NCT01889238) tested enzalutamide on patients 18 years or older that had locally advanced or metastatic AR-positive TNBC [64]. Patients that had prior treatments for advanced TNBC were eligible for the study; however, patients that had central nervous system metastases, cardiovascular diseases, or a history of seizures were excluded from this study. The patients were divided into two groups: the evaluable subgroup whose AR expression level was ≥10% and the intent-to-treat (ITT) subgroup which included all the patients involved. The results revealed that the PFS was 2.9 months in the ITT subgroup compared to 3.3 months in the evaluable subgroup. Additionally, the median OS was 12.7 months in the ITT subgroup compared to 17.6 months in the evaluable subgroup. Enzalutamide was well tolerated by most participants in this study. Fatigue was the only grade 3 or greater AE related to treatment, occurring in ≥2% of patients, with all other serious AEs being attributed to disease progression. This study is expected to be completed by December 2023.

Another phase II clinical study (NCT02750358) evaluated adjuvant enzalutamide treatment in patients with stage I–III AR-positive TNBC who had completed their standard-of-care treatment [65]. A total of 50 patients were initially enrolled in the study; however, only 35 patients completed at least one year of enzalutamide treatment to meet the trial’s endpoint for feasibility. Of the evaluated patients, the 1-year DFS, 2-year DFS, and 3-year DFS were 94%, 92%, and 80%, respectively. Enzalutamide was well tolerated in these patients and demonstrated low toxicity. The only grade 3 or higher AEs reported were fatigue (6%) and hypertension (2%). This study’s expected completion date is May 2024.

### 7.2. Enobosarm

Enobosarm is a non-steroidal selective androgen receptor modulator that, in AR-positive TNBC tumors, showed an ability to inhibit tumor growth [72]. In a phase II clinical trial (NCT02971761), a combination of enobosarm and pembrolizumab, an anti-PD-1 immunotherapy, was tested on AR-positive metastatic TNBC patients [66]. A total of 18 patients were initially enrolled in the trial and only 16 patients were evaluated for efficacy. This combinational therapy returned some clinical benefit, with 1 of 16 patients achieving a complete response, 1 of 16 patients receiving a partial response, and 2 of 16 patients with stable disease. Additionally, the response rate for this combination treatment was 13% and the clinical benefit rate (CBR) was 25% after 16 weeks. Enobosarm and pembrolizumab treatment was generally well tolerated, with the only grade 3 AEs reported being pain (6%), dry skin (6%), and diarrhea (6%).

### 7.3. Bicalutamide

Bicalutamide is a first-generation non-steroidal AR antagonist that is currently FDA-approved for the treatment of prostate cancer [73]. Bicalutamide binds AR through competitive inhibition, preventing its translocation to the nucleus and any further signaling. A phase II trial (NCT00468715) investigated bicalutamide’s efficacy and safety in ER-/PR-negative metastatic breast cancer patients that were highly expressing AR [67]. A total of 51 of the 424 (12%) screened patients tested positive for AR expression (≥10%), and 26 patients were evaluable for the study’s primary endpoint. In the evaluated patients, a 6-month CBR of 19% and a 12-week median PFS were achieved. Bicalutamide showed low levels of toxicity and no grade 4/5 AEs were reported. Grade 3 AEs related to elevated liver enzyme levels (aspartate aminotransferase, bilirubin, and alkaline phosphatase) were reported in one patient who had liver metastases, so it is unclear whether the events could be attributed to disease progression or to treatment. Otherwise, 1 of the 28 patients evaluated for safety reported grade 3 nausea.

Some researchers have begun exploring bicalutamide’s effectiveness in combination with other therapeutics for treating AR-positive TNBC patients. Particularly, the cyclin-dependent kinase (CDK)4/6-retinoblastoma pathway has been implicated in the progression of breast cancer, and some TNBC cell lines have demonstrated sensitivity to the use of CDK4/6 inhibitors [74]. An ongoing phase II clinical trial (NCT02605486) investigating bicalutamide in combination with CDK4/6 inhibitor palbociclib in AR-positive metastatic TNBC has demonstrated potential clinical benefit [68]. A total of 31 of the 33 enrolled patients were evaluated for the study’s endpoints. At the six-month mark, 11 of 31 patients were progression-free, and 10 of 31 patients had stable disease. The bicalutamide and palbociclib combination was fairly well tolerated by patients. The expected study completion date for this trial is November 2024. Another phase I/II clinical trial (NCT03090165) is investigating the use of bicalutamide in combination with the CDK4/6 inhibitor ribociclib in advanced AR-positive TNBC patients [69]. From the phase I clinical data, this combinational therapy has been well tolerated by patients and no unexpected toxicities have been reported. The study’s expected completion date is September 2024.

### 7.4. Seviteronel

Seviteronel possesses the ability to inhibit both AR and cytochrome P450 17α-hydroxylase/17,20-lyase (CYP17 lyase), the enzyme required for androgen production [75]. Additionally, seviteronel promoted the radiosensensitization of TNBC cell lines and decreased tumor volume when used in conjunction with radiation. An open-label phase I clinical study aimed to determine the appropriate dosage of seviteronel in women with ERα-positive breast cancer or TNBC, as well as its safety [70]. A total of 19 women were evaluated in this study, where 14 of the patients were classified as ER-positive while the other 5 were classified as TNBC. AR status was not considered at this time. Seviteronel was generally well tolerated by patients, with grade 3 or higher AEs being reported in only four subjects. Additionally, in the seven women given a 450 mg dose of seviteronel, the recommended phase 2 dose, four of the patients reached the 16-week CBR, with two of these patients being diagnosed with TNBC. Phase II of this clinical trial will expand the cohort to include men and women with either ER-positive breast cancer or TNBC.

## 8. Glucocorticoid Receptor

**Figure 4:**
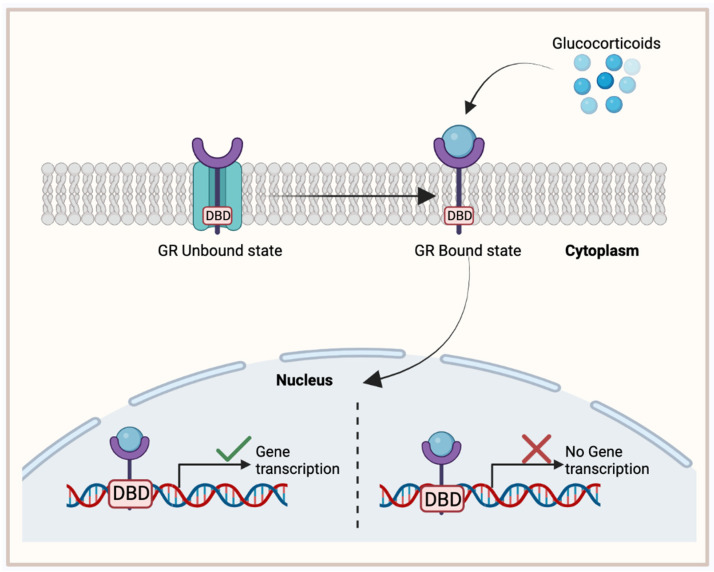
Glucocorticoid receptor (GR) binding mechanism, activating target gene expression. In its unbound state, GR is located in the cytoplasm with a chaperone complex. Once bound to its ligand, glucocorticoid, GR translocates to the nucleus where its DNA-binding domain (DBD) will activate or inhibit target gene expression. This figure was made using Biorender.com.

GR is a nuclear hormone receptor that is ubiquitously expressed and activated upon the binding of its respective ligand, glucocorticoid. Glucocorticoids are steroid hormones that are released by the adrenal glands and play significant and broad roles in metabolism, anti-inflammation, immune responses, and fetal development [76]. The GR structure is composed of a disordered amino-terminal domain (NTD), a DBD, and an LBD [77]. In its unbound state, GR resides in the cytoplasm, protected in a chaperone complex during its folding stages. However, once the glucocorticoid ligand binds to GR’s LBD, GR becomes activated and translocates to the nucleus where its DBD domain participates in specific DNA binding. Consequently, GR will then recruit various transcription factors that will further activate or repress target gene expression (Figure 4).

## 9. Glucocorticoid Receptor in TNBC

The expression levels of GR on TNBC cells vary considerably in different studies, ranging from 0% to 84% [78,79,80,81,82]. This discrepancy can be largely attributed to the lack of standardization among antibodies and methods used to detect GR on cells. Generally, the presence of GR on TNBC cells is associated with a poor patient prognosis and a worse OS [78,79]. This is in direct contrast with ERα-positive patients where the presence of GR corresponds to a better prognosis [83], suggesting that regulation of GR through ERα can heavily impact whether GR imparts a proliferative or antiproliferative functioning. TNBC patients with high GR expression are typically more resistant to chemotherapy-induced apoptosis, a phenomenon that is likely mediated through the GR-mediated upregulation of several pro-survival genes, including *MPK-1* and *SGK-1* [83,84]. In basal-like TNBC, GR and STAT3 bind the same regulatory region, cooperatively promoting the expression of hundreds of basal-like genes that are associated with cell proliferation, stemness, and the epithelial–mesenchymal transition (EMT) [85].

GR signaling in TNBC cells depends heavily upon its external environment. An abundance of TGFβ1 or cellular stress in the tumor microenvironment activates p39 MAPK signaling, resulting in ligand-independent but p38-dependent GR phosphorylation at Ser134 (pS134-GR), ultimately promoting TNBC invasion and anchorage-independent growth [86]. Additionally, pS134-GR promotes the expression of MAP_3_K_5_, an activator of p38 MAPK signaling, suggesting that GR participates in a feedforward loop in response to stressors present in the environment. Additionally, pS134-GR regulates several genes involved in metabolic reprogramming to favor cell migration [78], demonstrating GR’s essential role in metastasis.

Interestingly, GR signaling could possibly have a bi-faceted role in chemotherapy response. High GR expression in TNBC patients was associated with an increased responsiveness to anthracycline-based chemotherapy; however, it demonstrated a poor response to taxane-based therapy [87]. In ERα-negative patients who were given glucocorticoid alongside their anthracycline treatment, OS was improved, while ERα-negative patients treated with glucocorticoid alone showed a worse OS [88]. The mechanism of action behind GR’s interactions with anthracycline and taxane is not well understood; however, this observation allows for the potential use of GR as a biomarker for the outcome of different chemotherapies in TNBC.

## 10. The Clinical Use of RU486 (Mifepristone) in GR-Positive TNBC

RU486, otherwise known as mifepristone, is an antiprogesterone and anti-glucocorticosteroid agent that has a high affinity for PR and GR [89]. RU486 is capable of binding either receptor, maintaining them in an unfavorable conformation to inhibit any downstream signaling. RU486 is predominantly used to terminate pregnancy during the early developmental stages; however, preclinical data obtained in breast cancer cell lines and TNBC mice models suggest that it could function as a hormonal therapy as well [90,91,92,93]. Table 3 summarizes the results of clinical trials testing mifepristone in TNBC.

A randomized phase I clinical trial (NCT01493310) completed in 2018 was designed to determine the pharmacokinetics and toxicity of chemotherapy agent nab-paclitaxel (Abraxane, an albumin-bound nanoparticle formulation of paclitaxel) when used in combination with mifepristone in advanced GR-positive breast cancer patients [94]. A total of nine patients were enrolled in the trial, where six were diagnosed with TNBC. Of the six TNBC patients, two of them had a complete response, two had a partial response, one had stable disease, and one had progressive disease. Pharmacokinetic studies revealed that administration of mifepristone successfully delayed nab-paclitaxel clearance in a number of patients. Combinational treatment had manageable levels of toxicity, with some patients experiencing neutropenia as a result of this therapy.

A phase II, randomized, placebo-controlled clinical trial (NCT02788981) is investigating the use of nab-paclitaxel with or without mifepristone in patients with advanced GR-positive TNBC [95]. Of the 29 patients enrolled in the trial, 13 received nab-paclitaxel and a placebo, while the other 16 patients received the nab-paclitaxel and mifepristone combination. OS in the combination group was 9 months, while nab-paclitaxel alone was 6 months, though PFS was not significantly improved by the addition of mifepristone. The combinational treatment was generally well tolerated by patients, with the most reported grade 3 AE being neutropenia. This study is expected to be completed by August 2024.

A phase I/II clinical trial (NCT02014337) investigated the safety and efficacy of mifepristone in combination with eribulin, another FDA-approved chemotherapy agent for breast cancer, in patients with GR-positive TNBC [96]. Phase I included 16 patients with metastatic breast cancer while phase II had 21 patients with TNBC specifically. Across phase I and II, there were 23 evaluable patients at the recommended phase II dose (mifepristone 300 mg/day and eribulin 1.1 mg/m^2^). Following combinational treatment, three patients had a partial response, eight had stable disease, eleven patients had progressive disease, and one was still inconclusive. The median PFS was 9 weeks, which was generally better than the use of eribulin alone. In terms of safety, this combinational treatment was well tolerated, and the most commonly reported AE was neutropenia. Grade 3/4 AEs were limited to neutropenia, neuropathy, fatigue, hypokalemia, and nausea.

## 11. Conclusions

The inherent lack of distinct cellular targets and the pronounced heterogeneity of TNBC tumors has led to a significant deficiency in treatment options, resulting in a more challenging prognosis for TNBC patients. As a result, researchers have been exploring alternative therapeutic avenues for TNBC. Targeting steroid hormonal receptors, such as ERβ, GR, and AR, has shown some potential in inhibiting cancer cell proliferation and curbing tumor growth in biological models. These receptors could also be considered as biomarkers for determining patient prognosis and sensitivity to related treatments. Specifically, the presence of AR is associated with improved OS while GR is associated with a worsened outcome in TNBC, and patients expressing ERβ and mutant p53 had an increased responsiveness to tamoxifen treatment.

Clinical trials targeting these receptors in TNBC patients expressing high levels of ERβ, AR, or GR demonstrated moderate improvement of survival and patient outcome, particularly in patients with metastatic disease. The majority of therapies were well tolerated by patients, with a limited number of grade 3 or higher AEs. Thus, it appears that the use of these hormonal therapies could provide some benefit to a substantial proportion of TNBC patients.

## 12. Future Directions

While the current findings are promising, further research is needed to resolve gaps in the literature. Primarily, the majority of studies do not account for the heterogeneity of TNBC tumors. Subtype-dependent regulation of receptor signaling could partially explain the range of patient responses reported in clinical trials. Additionally, insight into the interactions between these hormone receptors is needed. While a small number of studies have reported on the crosstalk between the various hormone receptors [97,98,99,100], their mechanism of action is still largely unknown. Thus, further research regarding these hormonal receptors in TNBC could lead to a new effective treatment for some patients.

## Figures and Tables

**Table 1:** Ongoing or completed clinical trials targeting estrogen receptor beta in TNBC.

Trial (National Clinical Trial Identifier)	Phase		Condition	Interventions	Key Results	References
**Tamoxifen**			ERβ+/p53-mutant TNBC patient with brain metastases	Tamoxifen	Reduction in tumor volume in the brain metastases; currently, no signs of disease progression.	[36]
**Tamoxifen (NCT02062489**)	III	(1) (2)	ERα/PR-negative ERβ+ operable breast cancer patients	Adjuvant Tamoxifen	**No preliminary data available.** Study to be completed by May 2026.	[37]
**Toremifene** **(NCT02089854)**	IV	(1)	Patients with operable ERβ+ TNBC tumors	Toremifene + Anastrozole	**No preliminary data available.**	[38]
**17β-Estradiol (E2)**	II	(1)	Metastatic TNBC	E2	**Partial response**: 1/13 patients (Erβ expressing); little effect on **OS** and **PFS;** grade 3–4 **AE** in 4/17 patients; 2 cases of grade 3 dyspnea; 1 case of grade 3 vomiting; 1 case of grade 4 thromboembolism.	[39]
**17β-Estradiol (E2)** **(**N**CT03941730**)	II	(1)	Metastatic TNBC patients overexpressing Erβ	E2	**No preliminary data available.** Study to be completed by April 2024.	[40]

PFS: progression-free survival; OS: median overall survival; AE: adverse events.

**Table 2:** Ongoing or completed clinical trials targeting the androgen receptor in TNBC.

Trial (National Clinical Trial Identifier)	Phase		Condition	Interventions	Key Results	References
**Enzalutamide (NCT01889238)**	II	(1) (2)	Locally advanced or metastatic AR+ TNBC Intent-to-treat (ITT)—all patients AR expression ≥10%	Enzalutamide	**PFS**: (1) 2.9 months, (2) 3.3 months; **OS:** (1) 12.7 months, (2) 17.6 months; Fatigue (≥2%). Study to be completed by December 2023.	[64]
**Enzalutamide (NCT02750358)**	II	(1)	Stage I–III AR+ TNBC	Adjuvant Enzalutamide	**DFS:** 1-year: 94%; 2-year: 92%; 3-year: 80%; **Grade 3 or higher AEs related to treatment**: fatigue (6%), hypertension (2%). Study to be completed by May 2024.	[65]
**Enobosarm** **(NCT02971761)**	II	(1)	AR+ metastatic TNBC patients	Enobosarm + Pembrolizumab	**Complete response**: 1/16; **Partial response:** 1/16; Stable disease: 2/16; **Response rate** to combination treatment: 13%; **CBR**: 25% after 16 weeks; Grade 3 related AEs—pain (6%), dry skin (6%), diarrhea (6%).	[66]
**Bicalutamide** **(NCT00468715)**	II	(1)	ER–/PR– metastatic breast cancer patients highly expressing AR	Bicalutamide	**AR+ expression** (≥10%): 12%; 6-month **CBR**: 19%; **PFS:** 12-week median; Grade 3 AEs related elevated liver enzyme levels in one patient with liver metastases; Grade 3 nausea in 1/28 patients.	[67]
**Bicalutamide** **(NCT02605486)**	II	(1)	AR+ metastatic TNBC	Bicalutamide + Palbociclib	At 6-month mark: 35% **progression-free**; 32% **stable disease.** Study to be completed by November 2024.	[68]
**Bicalutamide** **(NCT03090165)**	I/II	(1)	Advanced AR+ TNBC patients	Bicalutamide + Ribociclib	**No preliminary data available.** Study to be completed by September 2024.	[69]
**Seviteronel**	I	(1) (2)	Women with ER+ breast cancer or TNBC 14/19: ER+ 5/19: TNBC	Seviteronel	AEs reported in only 4 patients; 7 women given 450 mg dose of seviteronel, 4/7 patients reached 16-week **CBR**, 2 of these patients diagnosed with TNBC. **Phase II trial** will expand cohort to include men and women with either ER+ or TNBC.	[70]

PFS: progression-free survival; DFS: disease-free survival; OS: median overall survival; CBR: clinical benefit rate; AEs: adverse events.

**Table 3:** Ongoing or completed clinical trials targeting the glucocorticoid receptor in TNBC.

Trial (National Clinical Trial Identifier)	Phase		Condition	Interventions	Key Results	References
**Mifepristone RU486 + Nab-paclitaxel** **(NCT01493310)**	I	(1)	Advanced GR+ breast cancer patients TNBC + patients	RU486 + nab-paclitaxel	**Complete response:** 2/6; **Partial response:** 2/6; **Stable disease:** 1/6; **Progressive disease:** 1/6; Some patients experienced neutropenia.	[94]
**Mifepristone RU486 + Nab-paclitaxel vs. Placebo** **(NCT02788981)**	II	(1) (2)	Advanced GR+ TNBC 13/29: Nab-paclitaxel + placebo 16/29: Nab-paclitaxel + RU486	Nab-paclitaxel + Placebo Nab-paclitaxel + RU486	**OS (2):** 9 months; **OS (1):** 6 months; **PFS:** not significantly improved by addition of RU486; **Grade 3 AE:** Neutropenia. Study to be completed by August 2024.	[95]
**Mifepristone RU486 + Eribulin** **(NCT02014337)**	I/II	(1)	Patients with operable GR+ TNBC	RU486 + Eribulin	**Phase I:** 16 patients with meta-static breast cancer; **Phase II:** 21 patients with TNBC; **Phase II dose combinational treatment partial response:** 3/23; **Stable disease:** 8/23; **Progressive disease:** 11/23; **Inconclusive:** 1/23; **Median PFS:** 9 weeks; **Grade 3/4 AEs:** neutropenia, neuropathy, fatigue, hypokalemia, nausea.	[96]

PFS: progression-free survival; OS: median overall survival; AEs: adverse events.

## Data Availability

All references cited in this review article are available in PubMed, ClinicalTrials.gov, or InTech.
